# Price, convenience, the buying experience, and other motivations for purchasing tobacco and e-cigarettes online

**DOI:** 10.18332/tid/152138

**Published:** 2022-09-05

**Authors:** Jessica L. King Jensen, Kayla Rebentisch, Hollie L. Tripp, Julie W. Merten

**Affiliations:** 1 Department of Health and Kinesiology, University of Utah, Salt Lake City, United States; 2 Franklin and Marshall College, Lancaster, United States; 3 Brooks College of Health, University of North Florida, Jacksonville, United States

**Keywords:** tobacco, e-tobacco, purchase behavior

## Abstract

**INTRODUCTION:**

Consumers have shifted to online purchases for many products, including tobacco and e-cigarettes. These shifts have occurred alongside internet tobacco purchasing restrictions being proposed and enacted across the US. The aim of this study was to identify motivations for and against purchasing tobacco and e-cigarettes online, to better understand potential impacts or loopholes.

**METHODS:**

We surveyed 463 US adults who reported ever purchasing tobacco or e-cigarettes in April 2021, using Amazon’s Mechanical Turk. Participants who reported purchasing tobacco or e-cigarettes online were asked to describe their reasons for doing so. Those who reported never purchasing online were asked to describe their reasons. Responses were triple-coded and thematically analyzed.

**RESULTS:**

Most respondents (n=330; 71.3%) had purchased tobacco or e-cigarettes online. We identified 14 reasons for purchasing tobacco or e-cigarettes online across four themes: price (cheaper online, discounts, bulk purchases, avoiding taxes), product characteristics (availability, quality), buying experience (convenience, time, COVID-19 concerns, avoiding shame, discretion, avoiding salespersons, reading reviews), and curiosity. We identified 13 reasons for not purchasing tobacco or e-cigarette products online across seven themes: buying experience (convenience, time, discretion, seeing the product), concerns (legality, safety, quality), consumption, price, supporting local, unaware, and uninterested.

**CONCLUSIONS:**

Both online and offline purchasers stated price and convenience motivated their choice to purchase tobacco or e-cigarettes online. Though few participants mentioned purchasing illicit products, concerns about legality and quality of online purchases were raised, and there was some awareness that online purchases attracted lower taxation.

## INTRODUCTION

Efforts to restrict internet tobacco purchases have been ongoing since the early 2000s. Following a series of commentaries and studies that identified easy access to online purchasing among youth^1-3^, companies entered into voluntary agreements to reduce online purchases of cigarettes. For example, in 2005, PayPal prohibited use of its platform to purchase cigarettes^4^, major credit card companies prohibited use of their cards for illegal sale of cigarettes online^5^, and FedEx, UPS, and DHL prohibited shipments of cigarettes to US consumers^6^. Despite these efforts, internet cigarette sales continued^7^. As a result, the US Congress enacted the Prevent All Cigarette Trafficking Act of 2009 (PACT Act), to address youth access and ensure consumer taxes are paid^8^. Specific provisions include prohibiting internet sales of cigarettes and smokeless tobacco to anyone under the legal age of sale, requiring vendors to verify age and identity at purchase and delivery, and prohibiting tax-free sales^8^. This legislation was extended to electronic cigarettes (e-cigarettes) in 2021^9^.

However, shifts in the nicotine product landscape, including the rise of e-cigarettes and other newer nicotine products, have led to increases in online tobacco and e-cigarette purchases. Between 2014 and 2017, estimates of usual purchase online increased from 2.5% to 4.4% among youth and 2.5% to 3.3% among adults^10^. Estimates of ever purchasing online are higher, with approximately 27.5% of adults in Canada, England, Australia, and the US, having ever purchased e-cigarettes online in 2016^11^. While more recent data on online tobacco or e-cigarette purchases are not available, we might expect sustained or increased prevalence due to two key factors. First, e-commerce sales in the US have increased at double-digit rates for the past two decades and have significantly outpaced brick-and-mortar retail growth^12^. Second, this study was conducted in April 2021, following widespread brick-and-mortar closures due to the global pandemic, which may have limited in-person tobacco and nicotine purchases and increased e-commerce^13,14^.

Few studies have explored the reasons for purchasing tobacco products online, but among those that have, the primary driver appears to be price^15,16^. One estimate indicated someone smoking a pack of cigarettes per day would save $1508 per year when buying cigarettes online compared to a retail store^17^. This substantial savings may be due to more internet cigarette vendors claiming a tax-free status^17^, or online vendors avoiding excise taxes for the respective delivery state^15^. The aim of this study was to identify motivations for purchasing tobacco and e-cigarettes online to identify potential loopholes or areas for additional policies or enforcement. With the potential for additional local, state, and federal regulations, the findings will have broad applicability for regulators.

## METHODS

In April 2021, we conducted a survey among a convenience sample of adults who reported ever purchasing tobacco or e-cigarettes using Amazon’s Mechanical Turk (MTurk). MTurk is an online marketplace commonly used for data collection in social science and tobacco control research^18,19^. Eligible participants included adults aged >21 years who resided in the US, reported ever purchasing tobacco or e-cigarettes, and had at least 50 tasks completed and an approval rating over 90%. Participants completed informed consent prior to beginning the 5-minute survey and received $1.25 through MTurk upon completion. The procedures for this study were reviewed and exempted by the University of Utah Institutional Review Board. Of 909 participants who initiated the survey, 875 completed the screening items, 743 met the survey eligibility criteria, 643 answered the question about purchasing online. Removing responses that were likely bots using best practices^20-22^ and through analysis of open-ended responses led to an analytic sample of 463 (72.0% of those who completed the survey; 62.3% of those eligible).

Participants were asked whether they had ever purchased tobacco or e-cigarettes online. Those who reported they had purchased tobacco or e-cigarettes online were prompted to ‘Describe reasons you purchase or have purchased tobacco or e-cigarettes online’. Those who had never purchased tobacco or e-cigarettes online were asked to ‘Describe below your reasons for not purchasing tobacco or e-cigarettes online’. Sociodemographic and tobacco use items included age, sex (male, female, other), race (select all: American Indian or Alaska Native, Asian, Black or African American, Native Hawaiian or Pacific Islander, White, Other), income (US$: <10000, 10000–14999, 15000–24999, 25000–34999, 35000–49999, 50000–74999, 75000–99999, 100000–149999, 150000–199999, ≥200000, and ‘I don't know or prefer to not say’), and past 30-day tobacco use (yes/no for cigarettes, cigars, e-cigarettes or vaping devices, smokeless tobacco, hookah or waterpipe, other).

We used descriptive analyses to characterize the sample. Thematic analysis was used to identify the motivations for and against purchasing online. After initial review of all responses to the open-ended items on reasons for/against purchase, 35 codes were identified. Three trained coders independently coded all responses (interrater reliability = 95.3%). Based on the data collection methods employed, coders used a verbatim approach to coding (coding what was there without interpretation). A fourth team member reviewed the results and determined the final code consulting with the full team in areas of discrepancy. Codes were sorted into categories and then into themes.

## RESULTS

Of 463 respondents, 330 (71.3%) reported ever purchasing tobacco or e-cigarettes online. Most participants were male (64.6%); 83.8% reported White race, 11.0% reported Black or African American race, 9.5% reported Hispanic ethnicity, 56.4% reported annual household income of ≥$50000, 74.5% reported past 30-day use of more than one nicotine product, with cigarettes (78.6%) and e-cigarettes (67.8%) most prevalent, and the mean age was 37.0 (SD=10.4) years.

In qualitative analyses, we identified 14 reasons for purchasing tobacco or e-cigarettes online across four themes and 13 reasons against purchasing tobacco or e-cigarette products online across seven themes. Table 1 includes sample responses for each of the reasons identified for purchasing products online. Table 2 includes sample responses for each of the reasons identified for not purchasing online.

**Table 1:** Reasons for online purchases among consumers who purchased tobacco or e-cigarettes online (N=330)

*Theme*	*Category*	*Responses n (%)*	*Example quote*
**Price**	Lower cost online	96 (29.1)	There’s only one reason to buy tobacco products online and that is because they are cheaper than what I can buy at a local store.
	Discounts and promotions online	35 (10.6)	They were doing a sale, buy 3 and get the 4th free, so it seemed to be a good deal and better than what I would be able to find if I went to a physical store.
	Buy in bulk	4 (1.2)	It is (or was, up until last month) the only place to buy bulk ingredients to make my own vapor liquid.
	Avoid taxes	3 (0.9)	I have purchased tobacco online because the selection is wider and the prices are cheaper when purchasing it in bulk. I can have the convenience of having it shipped to my mailbox and also there is no sales tax when purchasing from out of state.
**Product characteristics**	Availability		
	General	67 (20.3)	I wanted to try others that were not available to me around my area.
	Flavors	20 (6.1)	I figured I would find a wider selection of flavors and products by searching online than going to the handful of physical stores in my state.
	Nicotine	2 (0.6)	I could not get the exact nicotine mod in person.
	Contraband product	1 (0.3)	Wanted contraband clove cigarettes.
	Product quality	15 (4.5)	It is very safe and good quality of products and price also very discount.
**Buying experience**	Convenience	91 (27.6)	Buying online can be very simple and easy, as you just order them and your item will arrive at your designated address without heading out to store. Just set a reminder for your agenda to order them when you are low on supply.
	Save time	26 (7.9)	It was a time saving method.
	COVID-19-related	37 (11.2)	There was a Lockdown order in my area and I needed to smoke, so I ordered it online.
	Avoid shame	3 (0.9)	Sometimes people often talk about us like we are tobacco addicts. So sometimes I bought it on online.
	Discretion	7 (2.1)	Purchasing online is maintaining my privacy.
	Avoid salespersons	7 (2.1)	Cheaper, don’t have to deal with insufferable employees in vape/tobacco shops.
	Read reviews	7 (2.1)	It was less intimidating to purchase an e-cigarette online when I was new to the process. I was nervous to go into the store without knowing what I was doing, lingo to use, what type of product I might want, etc. I could also read reviews and do research without worrying about being pressured or up-sold.
**Curiosity**		3 (0.9)	I purchased e-cigarettes online for trial purpose only. I just want to know how benefitable it was to purchase through online. Also during pandemic I don't want to go away from my home. So I purchased through online.

Responses have not been edited from the text provided by respondents; spelling and punctuation errors may exist. Because responses could fall into multiple categories, the total N is greater than 330; 85 (25.8%) responses were about e-cigarettes, 20 (6.1%) cigarettes, 2 (0.6%) roll-your-own, and 1 (0.3%) cigars. Percentages are presented to provide additional context to the reader, but they should not be interpreted to represent population-level prevalence as these were responses to an open-ended question to a convenience sample. Prevalence may have differed if participants were asked to endorse from a list or the study was conducted among a population sample.

**Table 2:** Reasons against online purchases among consumers who had never purchased tobacco or e-cigarettes online (N=133)

*Theme*	*Category*	*Responses n (%)*	*Example quote*
**Price**	Cheaper in person	5 (3.8)	I've looked for deals online for ryo tobacco but I've never seen better prices than local stores.
**Buying experience**	Convenience	22 (16.5)	I just can get it from the shop easily.
	Save time	13 (9.8)	Easier just to go to the store to buy and not have to wait for a delivery.
	Discretion	1 (0.8)	I would not want my family to know I smoke.
	Receive guidance or see/touch the product	16 (12.0)	I normally just buy them from a local retailer because then I can ask questions and actually handle the product myself.
**Boost local economy**		1 (0.8)	I just like going to my local store. I feel like I’m contributing to local economy.
**Consumption**	Don’t consume often	6 (4.5)	I usually like to purchase only one packet and it doesn't make sense ordering single items online.
	Limit consumption	1 (0.8)	I'm not sure what the legality of it is due to state taxes. Also, I try not to have too many nicotine products at one time or else I feel like I will use them excessively.
**Concerns**	Legality	8 (6.0)	I have tried but everywhere I go for chewing tobacco seems like they don't sell it to me. Probably because of the state that I live in. I would love to be able to purchase online though.
	Safety	2 (1.5)	I don't trust most websites.
	Quality	12 (9.0)	I would be skeptical about purchasing tobacco online, especially since I have never done it before. I would be afraid that the company would not be reputable or that something was put into it. That would just be weird.
**Unaware**		18 (13.5)	I didn't even know this was an option.
**Uninterested**		48 (36.1)	I have no reason to, I usually just go to the gas station.

Responses have not been edited from the text provided by respondents; spelling and punctuation errors may exist. Because responses could fall into multiple categories, the total N is greater than 133; 10 (7.5%) responses were about e-cigarettes, 20 (15.0%) cigarettes, 5 (3.8%) cigars, and 1 (0.8%) smokeless tobacco. Percentages are presented to provide additional context to the reader, but they should not be interpreted to represent population-level prevalence as these were responses to an open-ended question to a convenience sample. Prevalence may have differed if participants were asked to endorse from a list or the study was conducted among a population sample.

### Reasons for purchasing online

We identified 14 reasons for purchasing tobacco or e-cigarettes online across four themes: price (cheaper online, discounts, bulk purchases, avoiding taxes), product characteristics (availability, quality), buying experience (convenience, time, COVID-19 concerns, avoiding shame, discretion avoiding salespersons, reading reviews), and curiosity. Further details on the 14 reasons for purchasing tobacco or e-cigarettes online are presented by theme.

Price-related reasons included lower costs, the ability to use discounts and promotions, the ability to buy in bulk, and to avoid taxes. For example, one purchaser stated: ‘vapes were cheaper online and had good discounts’.

Product characteristics included availability and quality of products purchased online. Consumers reported broader availability online, including generally and for flavors and nicotine: ‘there are better varieties and flavors’. Based on the prevalence of comments on product availability, the availability category was further subdivided into flavors, nicotine, and contraband products (Table 1). Participants who had purchased online mentioned the product quality was good, with none of the respondents mentioning concerns about product quality.

Buying experience-related reasons refer to the process of purchasing online and include convenience, saving time, COVID-19 concerns, to avoid shame, because it is discreet, to avoid salespersons, and to read customer reviews. COVID-19 concerns included statements like: ‘I started doing it because of Covid. It's more because I am buying all kinds of things online that I have never bought before’. Several experience-related reasons revolved around the stigma associated with using tobacco products, including to avoid shame: ‘Purchasing tobacco online makes me less exposed to people seeing me public as a smoker’, and to avoid salespersons: ‘because there are no curious people watching me while I do my shopping’.

The final theme identified for purchasing tobacco or e-cigarettes online was curiosity. Three participants noted they tried purchasing online out of curiosity or because they ‘Just wanted to try’.

### Reasons for not purchasing online

We identified 13 reasons for not purchasing tobacco or e-cigarette products online across seven themes: buying experience (convenience, time, discretion seeing the product, (legality, safety, quality), consumption, price, to support the local economy, unaware, and uninterested. The most common reason was a lack of interest, noted by 36.1% of participants who reported never purchasing tobacco or e-cigarettes online. The 13 identified reasons for not purchasing tobacco or e-cigarette products online are discussed further by theme.

Buying experience again refers to the process of purchasing online or in person and includes convenience, saving time, wanting discretion, to receive guidance or to see/touch the product. Similar to reasons for purchasing online, participants who had not done so noted that purchasing in person was convenient and saved time. Participants also preferred to see/handle the product before purchasing: ‘I also like the experience of looking over various cigars and selecting some’. One participant preferred to purchase in person to support their local economy.

Concerns included expressed hesitation due to legality, safety, and quality. The legality of purchasing online was mentioned by a few participants, including product preferences (e.g. cigarettes not available for purchase online) and location (e.g. state restrictions). Some expressed quality concerns about the product: ‘I would be skeptical about purchasing tobacco online, especially since I have never done it before. I would be afraid that the company would not be reputable or that something was put into it’.

Consumption-related reasons included not smoking that often and to control smoking. Each of these implied that purchasing online meant purchasing more product. With regard to price, participants noted that the internet was more expensive than purchasing in person or that hidden charges might be applied online: ‘Additional charges and additional dissatisfaction. Online buying has extra charges that are not included while ordering for the product’. The final two reasons were that participants were unaware they could purchase online or were not interested in doing so.

## DISCUSSION

We surveyed a convenience sample of 463 US adults who had ever purchased tobacco or e-cigarettes and identified 27 motivations for and against purchasing products online. Several themes were consistent across those who had and had not purchased online, including price, convenience, and buying experience. The most common reasons stated for purchasing online were lower price, convenience, and product availability, while the most common reason for not purchasing online was a lack of interest or need.

Price was a prominent theme, with participants highlighting the low prices, discounts and promotions, the option to buy product in bulk, and to avoid taxes as reasons for purchasing online. These findings align with prior work examining factors related to online cigarette purchases^15,23^. Increasing price has long been an effective strategy to reduce tobacco use^24^, though the effectiveness relies on the price increase being passed on to consumers. While only a few noted avoiding taxes, it is possible this might be a more prevalent response if participants were given a list of reasons to which they could endorse agreement. We also found that those who had not purchased online noted price as a reason to purchase in person. In this case, participants noted that hidden costs and shipping fees often lead to higher prices than purchasing in person. E-commerce research has noted the increased price sensitivity among online shoppers due to the feasibility of price comparisons^25^. Tobacco and e-cigarette discounts and promotions have been restricted in some brick-and-mortar retailers^26,27^, which may account for the motivation of discounts identified in this study. E-commerce research also indicates consumers often purchase more items and spend more money per transaction compared to in person^28^, which may be attributed to impulse buying that is more likely to occur online^25^. Adults who smoke or vape more are more likely to purchase their tobacco or nicotine online^29,30^, which might be why participants reported wanting to limit their consumption or that they did not consume enough, as reasons for not purchasing online.

The buying experience was a major theme, with numerous categories identified for both groups. Convenience and time were reasons for both purchasing online and in person. This justification appears based on preference regarding time spent purchasing versus receiving the product, and similar findings have been noted for purchasing medication online^31^. Additional motivations for purchasing in person included the experience of seeing, smelling, and touching the product, as well as receiving guidance from store employees. Conversely, some noted that they preferred to purchase online to avoid store employees. This was similar to responses of discretion and avoiding shame, which have also been noted as reasons for purchasing prescription medications online^32^. The PACT Act intended to make the sale of online cigarettes less convenient with its passage in 2009^8^, but online sales continued. The problem may be attributed to the difficulty of shippers to ascertain – or establish reasonable cause – as to whether a package contains prohibited products. If the seller does not disclose this information, the shipper may be unable to abide by the rules of the PACT Act, thus enabling the shipping of tobacco to remain convenient.

The survey was conducted one year after COVID-19 restrictions were enacted across the US, and several participants noted this as a reason for purchasing products online. As rates of e-commerce have been shown to have increased during the pandemic^13,14,33^, we expected similar findings here, though the magnitude is unclear due to the nature of this study. Concerns noted among those who had not purchased online related to legality, safety, and quality. Website trust and online safety concerns are prevalent across e-commerce sites^25,34,35^, and legality concerns have been noted for purchasing medications online^32^. It is unclear whether individuals who had purchased online were concerned about online safety or legality, though some responses suggest illegal activity occurs (e.g. purchasing contraband product). Improved communication to retailers or enforcement among online retailers may help ensure regulations are followed and may also serve to protect consumers who may inadvertently purchase illicit product. This may also indicate a need for campaigns to inform consumers about the legality of online tobacco purchases.

### Limitations

There are several limitations to note. We surveyed a convenience sample of participants recruited through MTurk. The high rates of online purchase among participants in this sample may not be generalizable to the US population; an analysis of 2016 e-cigarette purchases indicated 27.5% had ever purchased online^11^, which is lower than the 71.3% reported in the present study. We employed best practices^20-22^ to improve data quality as well as reviewed each open-ended item for inclusion, but it is possible that some bots were included. Due to the self-reported nature of data collection, social desirability bias may have impacted some responses, which might have resulted in fewer participants reporting illicit behavior. Additionally, data were collected as part of a larger study examining tobacco use behaviors, which limited the number of items due to concerns for participant burden. Participants were asked only one question about their internet purchasing, based on whether they had purchased online or not. We did not have data on which products were purchased online, which would better inform conclusions around legal purchase behavior or differences in behavior by product purchased. We attempted to examine responses by product use to determine whether reasons differed by products legal to purchase online, but the majority of participants reported use of multiple products, including both e-cigarettes and cigarettes. The reasons for purchasing online appear more focused on e-cigarettes and those against appear to pertain to cigarettes, but we are unable to make further inferences. Because dual and polytobacco use are common^36^, future studies may further examine the influence of polytobacco use and demographic characteristics on motivations for purchasing tobacco and e-cigarettes online. Despite these limitations, we identified an extensive list of motivations for and against purchasing products online.

## CONCLUSIONS

Both online and offline purchasers stated that price and convenience motivated their choice to purchase tobacco or e-cigarettes online. Though few participants mentioned purchasing illicit products, concerns about legality and quality of online purchases were raised, and there was some awareness that online purchases attracted lower taxation. Potential educational opportunities were identified for both retailers and consumers, as well as areas for potential enforcement. We recommend further studies with nationally representative samples to provide population estimates of motivations driving online purchases. In addition, studies are needed to compare online and offline pricing, as respondents claimed both were cheaper, as well as to examine whether online sales result in lower prices due to tax evasion or avoidance.

## Data Availability

The data supporting this research are available from the authors on reasonable request.
